# 

**Published:** 1889-05-04

**Authors:** 


### Amusements and Relaxation.

### Word Competition.

Commenced April 6th, Ends June 29th.

Three Prizes of 15s., 10s., 5s., will be given for the largest number of words derived from the words set for dissection.

Proper names, abbreviations, foreign words, words of less than four letters, and repetitions are barred; plurals, and past and present participles of verbs, are allowed. Nuttall's *Standard* dictionary only to be used.

N.B.—Word dissections must be sent in WEEKLY, arranged alphabetically, with correct total affixed.

The word for dissection for this, the *Fifth*, week of the quarter being

"E D I N B U R G H."

### Results of Third Week.

| Names. | April 25. | Totals. | Names. | April 25. | Totals. |
|---|---|---|---|---|---|
| Patience | 132 | 369 | Tiny | 125 | 296 |
| Nurse Duty | 131 | 362 | N'importe Qui | 112 | 283 |
| Lightowlers | 126 | 359 | Pollie | 136 | 277 |
| Scrooge | 133 | 368 | W. G. R. | 115 | 257 |
| Reldas | — | 232 | Robe | — | 150 |
| Tinie | 139 | 372 | Beryl | 101 | 242 |
| K. G. | 123 | 344 | Nesta | 33 | 124 |
| Broxbourne | 132 | 350 | Daffodil | — | 89 |
| Lauriston | 126 | 351 | A Schoolgirl | — | 117 |
| Spes | 131 | 351 | Montague | — | 79 |
| Gretta | — | 224 | Sheeny | 33 | 111 |
| Francesca | 131 | 346 | Alice R. | — | 63 |
| Heatherbell | — | 185 | Belfast | 105 | 163 |
| Sycamore | 52 | 201 | Gentian | 139 | 192 |
| Arctus | 116 | 315 | Hope | — | 39 |
| Amicus | 138 | 339 | Louie | 128 | 128 |
| Scratcher | 54 | 197 | Hamilton | 121 | 121 |
| Jemima | — | 138 | E. M. | 120 | 120 |
| Concours | — | 177 | F. J. D. | 112 | 112 |
| C. J. S. | — | 134 | Ladyr | 102 | 102 |
| See-saw | 130 | 313 | Surgical Nurse | 99 | 99 |
| Rosemont | — | 173 | Violet | 95 | 95 |
| Jenny Wren | — | 126 | L. Pearce | 32 | 32 |
| Eric | 119 | 294 | | | |

### Notice to all Correspondents.

N.B.—All letters referring to this page which do not arrive at 139 and 140, Salisbury-court, London, E.C., by the *first post on Thursdays*, and are not addressed PRIZE EDITOR, will in futu ıe be disqualified and disregarded.j

### Scraps and Gleanings.

A case of smallpox has occurred at Aberdeen.

Hospital Sunday will be held at Norwich on June 16th.

Dr. Richard Daly has been elected dispensary doctor for Ringaskiddy.

Miss Emily Faithful has been granted a pension of £50 from the Civil List.

Dr. Suckling has been appointed medical officer of Birmingham Infirmary.

The late bazaar at Merthyr resulted in a sum of £77 handed over to the hospital.

A Cottage Hospital is to be erected as a memorial of the late Duchess of Sutherland.

A meeting of ladies has been held at Lancaster in aid of the infirmary and dispensary.

Mr. C. S. Johnston has been appointed assistant medical officer of Birmingham Infirmary.

Dr. A. Wilson has been presented with a marble clock by the Mid-Calder Ambulance Class.

The death is announced of Dr. Howard, of Montreal, one of Canada's most distinguished physicians.

Mr. Trowbridge, a West Bromwich chemist, has been fined 5s. for refusing to have his child vaccinated.

Mr. F. R. A. Taylor has taken the Llewellyn Scholarship and Governors' Clinical Gold Medal at Charing Cross.

Mr. Tuck has been appointed surgeon-dentist of Pembrokeshire Infirmary, in place of Mr. Helyar, resigned.

Columns of the *Dunfermlin Journal* are taken up with letters on the subject of a proposed hospital for the town.

An old woman, named Payne, has just died at Faversham Workhouse, aged 104. She had been married four times.

Birmingham Skin and Lock Hospital treated 2,200 patients last year. The income exceeded the expenditure by £280.

The death is announced of Dr. Alexander Harvey, late Emeritus Professor of Materia Medica at Aberdeen University.

Cornelius Jordah, a boy of 13, has died in St. Thomas's Hospital. The boy ran a nail into his foot, and lockjaw resulted.

The instruction of Prof. Percy Frankland at Dundee will be recognised in future by the Medical Board of Edinburgh University.

Lady Dufferin will preside at the prize-giving to successful students of the London School of Medicine for Women on June 25th.

Mr. W. J. Penny, of King's College, has been elected surgeon of Bristol General Hospital in place of the late Mr. W. P. Keall.

The drainage of Dublin Barracks has again been overhauled by Prof. Hull and Mr. Field, and they cannot find cause for the continued fever.

The promoters of the scheme for supplying a hospital for Wallsend are holding almost daily meetings in the workshops to arouse interest in the movement.

Great interest has been aroused at Norwich by the news that Mr. W. Cadge is the anonymous governor who, eight years ago, promised £10,000 to the hospital.

The Convalescent Home at Dover, recently given by Mrs. Charlotte Rusher for the use of certain friendly societies, is open to the reception of all members of such societies.

The Vestry of St. Pancras has made a donation of £100 to the building fund of the New Hospital for Women, out of a sum they have at their disposal for charitable purposes.

The birthday of the Mission to Deep-Sea Fishermen will be kept on May 15th, when a large public meeting will be held at Exeter Hall. Birthday gifts will not be refused.

Mrs. Morris (Florence Terry) takes the place of her sister Marion in the series of theatricals organised for the benefit of the Ancoats Hospital, Manchester, April 24th to 27th.

The matron of Carmarthenshire Infirmary appeals for wild flowers for the wards. Now that the woods are carpetted with these beautiful blossoms, no hospital ward should remain undecorated.

The investigation into allegations made with reference to a boy, named Mulroy, who died lately at Jervis-street Hospital, Dublin, has resulted in the complete exoneration of all the hospital authorities.

Mr. Shelmerdine has been presented with a piece of silver plate in recognition of his services as chairman to the Salford Royal Hospital. A portrait of Mr. Shelmerdine has also been presented to the hospital.

The Countess of Meath, on behalf of the Kyrle Society, makes an earnest appeal for more ladies to help in giving bright, varied, and high-class entertainments in the workhouses and hospitals in the metropolis.

The Hackney Board of Guardians have decided not to take any proceedings against the persons reported to them by the vaccination officer of the eastern district with a view to enforcing the provisions of the Vaccination Acts.

The will of Mrs. Emily Bulmer, late of Strood, Kent, widow, who died on March 5th, was proved on April 4th. The testatrix gives £1,000 each to the British Home for Incurables (Clapham-rise), and St. Bartholomew's Hospital (Rochester).

Both the lady doctors in Bombay have just taken to themselves husbands. Dr. Edith Peachey's better half, besides being a wealthy wine merchant, is also hon. secretary for Lady Dufferin's Fund, and an enthusiastic naturalist.

The last entertainment of the twenty-second season took place on the 16th ult. at the Brompton Hospital, when Rossini's "Stabat Mater," under the direction of Mr. William Carter, assisted by Mr. Churchill Sibley, formed the programme.

The following members of the medical profession will probably be nominated to serve upon the Royal Commission of Inquiry into the Working of the Vaccination Acts: Sir Andrew Clark, Bart., President of the Royal College of Physicians; Sir James Paget, Bart.; Mr. Savory, President of the Royal College of Surgeons; Prof. Michael Foster; and Dr. Creighton.

